# Study protocol: post-harvest losses of fruits and vegetables and their relationship with the nutritional status of women and children

**DOI:** 10.3389/fpubh.2025.1654786

**Published:** 2025-09-18

**Authors:** Benedicta Twum-Dei, Herman Erick Lutterodt, Reginald Adjetey Annan, Linda Nana Esi Aduku

**Affiliations:** ^1^Department of Biochemistry and Biotechnology, College of Science, Kwame Nkrumah University of Science and Technology, Kumasi, Ghana; ^2^School of Nursing and Midwifery, Heritage Christian University College, Accra, Ghana; ^3^Department of Food Science and Technology, College of Science, Kwame Nkrumah University of Science and Technology, Kumasi, Ghana

**Keywords:** post-harvest loss, fruits and vegetables, women, children, nutritional status

## Abstract

**Introduction:**

Sub-Saharan Africa loses up to 50% of fruits and vegetables produced annually, with Ghana alone reporting post-harvest losses ranging between 30 and 50% across the value chain. These losses significantly threaten nutrition security by reducing the availability of essential nutrients in the food system. Capturing these lost nutrients is crucial to promoting healthy and affordable diets. This study protocol presents a community-based randomised controlled trial (RCT) being implemented in the Ho West District of Ghana. The trial aims to investigate the association between post-harvest loss (PHL) of fruits and vegetables and the nutritional status of women and children, while also identifying loss drivers across the value chain.

**Methods:**

This experimental trial study employs an untreated control group design and involves 280 participants, comprising 70 farmers, 70 traders, and 140 women and their children. Socio-demographic data and post-harvest practices will be collected from all participants to estimate nutritional losses. Dietary intake of fruits and vegetables will be assessed using food frequency questionnaires, while food consumption and household hunger will be measured using validated scoring tools. Anthropometric and nutritional measurements will be recorded for women and children using WHO protocols. Women in the intervention arm will receive nutrition education and counselling focused on storage and preservation strategies to reduce food waste.

**Potential impact:**

This study will provide evidence on the link between post-harvest loss and nutritional outcomes and inform practical interventions for reducing PHL at the community level. It offers a platform for building capacity among farmers, traders, and caregivers through increased awareness and behaviour change. By integrating nutrition education into PHL mitigation, the findings have the potential to influence food systems policy, improve household nutrition, and contribute to global efforts to reduce food loss. The trial is currently ongoing, and this protocol outlines the study’s design, methodology, and implementation framework.

**Clinical trial registration:**

This trial is registered with the Pan African Clinical Trial Registry (PACTR202310582275172).

## Introduction

Post-harvest loss (PHL) is defined by the Food and Agriculture Organisation (FAO) as the measurable reduction in both the edible mass (quantity) and nutritional value (quality) of food intended for human consumption (1). The post-harvest system includes several interconnected processes that begin at harvest and extend through processing, marketing, preparation, and ultimately, household consumption. At each of these stages, substantial food losses occur globally, resulting in decreased food availability and accessibility (2, 40).

Globally, an estimated one-third (1.3 billion tonnes) of food produced for human consumption is lost or wasted annually (1). These losses are particularly severe in developing countries, where they can lead to income reductions of at least 15% for an estimated 470 million smallholder farmers and others along the food value chain (3, 4). Most of these losses are attributed to food loss along the value chain, which accounts for approximately 90% of total waste (5). This has far-reaching implications for food prices, food security, and nutritional health (38).

In Sub-Saharan Africa and other low-income regions, plant-based foods form the key source of household diets, supplying nearly 90% of daily energy and 80% of protein requirements (5). However, these food categories, including grains, fruits, vegetables, legumes, and tubers, represent up to 92% of all post-harvest losses in the region. Among them, fruits and vegetables experience the highest losses, with estimates suggesting that nearly 45% are discarded or wasted before reaching consumers (6, 7).

Post-harvest loss of fruits and vegetables is a significant concern for achieving food security and reducing global hunger (8, 9). In Ghana, approximately 30–50% of fruits and vegetables are lost along the entire value chain (10–12). These losses occur at different stages, including harvesting, handling, storage, and retail. A study in Ethiopia showed that post-harvest handling, storage, and marketing account for 85.6, 11.6, and 2.8% of total losses, respectively (46, 48). As a result, there is a decreased availability of essential, nutrient-rich foods, especially for vulnerable groups such as women and children, who have high nutritional needs (13). The shift towards less nutritious, processed, or starchy foods due to limited access to fresh produce further worsens micronutrient deficiencies (14, 15).

This study addresses a critical public health issue: the extent to which post-harvest losses of fruits and vegetables contribute to nutritional deficiencies in women and children. A growing body of data supports the underlying causes of nutritional deficiencies. However, understanding a long-term solution to this enduring issue would require considering the complex interactions between agricultural and health factors, especially through nutrition-sensitive agricultural interventions that simultaneously address food system and health challenges (16). While many studies have examined PHL at the farm and retail levels (11, 17, 18), few have explored its magnitude and nutritional implications at the household level (19). Moreover, there remains a lack of systematic evidence examining the direct relationship between post-harvest losses and nutritional outcomes.

This study tackles a significant gap in understanding how post-harvest losses of fruits and vegetables contribute to nutritional outcomes among women and children in low-resource rural areas. While earlier research has looked at losses at the farm or retail level, few have focused on household-level losses or directly connected them to measurable nutritional outcomes. Therefore, this study presents a community-based intervention study aimed at estimating post-harvest losses along the value chain and assessing their impact on the nutritional status of women and children. It also aims to determine how structured nutrition education and counselling can minimise losses and enhance nutritional outcomes, thereby directly addressing the identified issue. By engaging key actors along the value chain and households, this study contributes to ongoing efforts to strengthen food system resilience, public health, and nutrition security.

## Study objectives

The primary objective of this study is to investigate the relationship between post-harvest losses of fruits and vegetables and the nutritional status of women and children in the Ho West District of Ghana. Specifically, the study aims to estimate the extent and drivers of post-harvest losses at the farm, retail, and household levels, to determine the nutrient losses associated with post-harvest waste of fruits and vegetables, to examine the relationship between the nutritional status of women and children in households and fruits and vegetables losses and to evaluate the effect of a structured community-based nutrition education and counselling intervention on post-harvest practices, fruit and vegetable consumption, and nutritional outcomes.

## Research design and methods

### Study setting

The study will be conducted in the Ho West District of the Volta Region in Ghana. The Ho West district shares borders with the Republic of Togo, and its capital, Dzolo-Kpuita, is located 175 km northeast of Accra. The total population is 95,000 (20). Anaemia and undernutrition remain critical public health issues in this region. Recent national trends indicate that anaemia prevalence among children under 5 years, although decreasing, remains alarmingly high at 48.9% as of 2022, down from 76.1% in 2003 (21). In the Volta Region specifically, hospital-based data shows that over 55% of children under five are anaemic (22). Stunting, a marker of chronic undernutrition, also affects nearly one in five children under five in Ghana (23). In the Ho West district, 73.5% of the people work in agriculture in some capacity. Less than 1 % (0.06%) of households involved in agriculture are involved in fish farming, with the majority (95.2%) in crop farming (47). Farmers in this region cultivate a diverse range of fruits and vegetables, including bananas, pineapples, mangoes, pears, and oranges. These four farming communities, Abutia Kissiflui, Dededo, Amedzofe, and Kpedze, will be the focus of the study. The district’s busiest market, located in Abutia Kissiflui, generates up to 75% of the district’s tax revenue. Contributions from Dededo, Amedzofe, and Kpedze are 12.3, 8.7, and 4%, respectively. Kpedze has a population of 4,692 people, of which 12% are children under the age of five and 51% are women. There are 3,360 people living in Dededo, 16% of whom are children under five, and 53.3% of whom are women. A total of 1,561 people reside in Amedzofe, with 51.2% of them being women and children. With 1,667 residents, Abutia Kissiflui has a female population of 54% and a child population of 13.5% (20, 24). The district’s southernmost neighbourhoods, Kpedze and Amedzofe, were selected for the intervention group, while the northernmost neighbourhoods, Dededo and Abutia Kissiflui, were designated as the control group. Out of the 14 towns in the district, these towns were specifically chosen. To prevent contamination, the control and intervention groups were selected based on their proximity to one another and their geographic location. Also, data on nutritional treatments and services provided in each group will be recorded.

### Study design

The study is an experimental trial with an untreated control group, adhering to the Consolidated Standards of Reporting Trials (CONSORT). The study will be conducted in three main phases: Phase I (problem identification and quantification), Phase II (intervention development and implementation) and Phase III (evaluation of intervention). The outcome of the study will be assessed twice, at baseline and post-intervention. The intervention will be tested on households, which will receive structured education on fruit and vegetable intake, as well as methods for waste and food preservation. Women in the household will be educated on food preservation methods and how to store various types of fruits and vegetables at home. They will receive intervention for 6 months.

### Study duration

The study will have a total duration of 16 months. Recruitment of participants will last for 1 month. Data collection on anthropometric indices, blood samples, and dietary intake will be conducted over 3 months. The intervention phase will last for 6 months. A duration of 3 months will be used for the statistical analysis and final write-up.

### Study status and timeline

**Figure 1:**
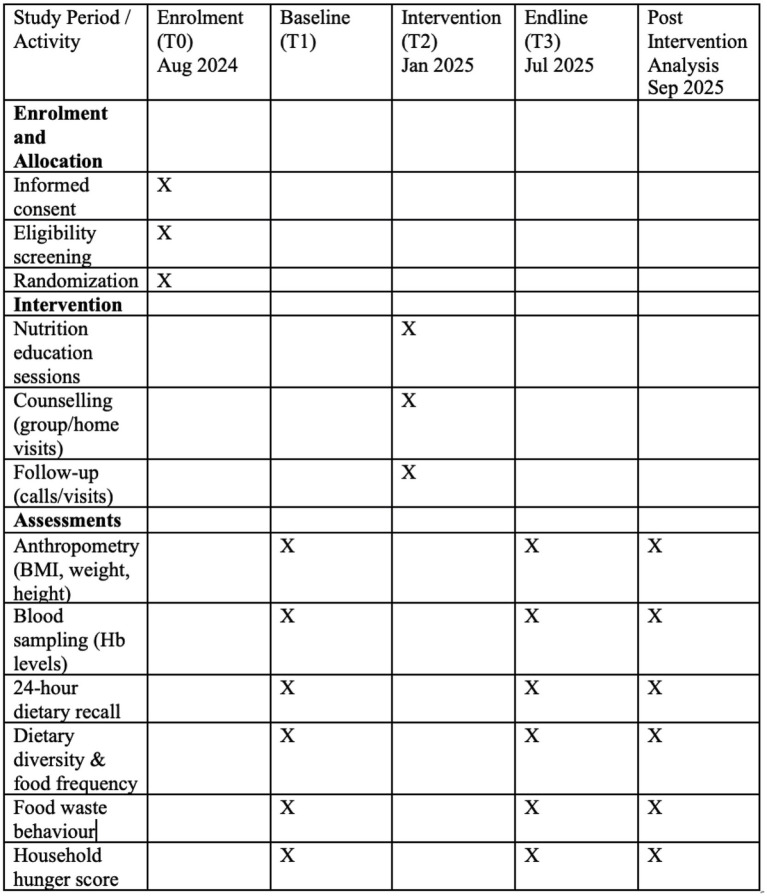
SPIRIT schedule of enrolment, interventions, and assessments [Adapted with permission from Chan et al. (49)].

This protocol outlines a community-based randomised intervention study aimed at improving nutritional outcomes and reducing fruit and vegetable waste. The study commenced on 1st August 2024 and is expected to conclude on 31st July 2025. At the time of this submission, Participant recruitment has been completed, and data collection is ongoing, with plans to continue through to 31 July 2025. Data analysis and result generation will commence after the conclusion of data collection, with preliminary results anticipated by September 2025. No results have been generated or analysed prior to this submission. The study timeline, including enrolment, intervention, and assessment time points, is presented in Figure 1 following the SPIRIT guidelines (SPIRIT 2013 Statement).

### Sampling

A multistage sampling approach will be employed to select study participants. First, the Ho West District in the Volta Region of Ghana will be purposively selected due to its significance in fruit and vegetable production, retail activities, and rural household participation. Second, the district will be stratified geographically into northern and southern zones. From each zone, two communities (Amedzofe and Kpedze in the north; Abutia-Kissiflui and Dededo in the south) will be purposively selected based on agricultural diversity and accessibility.

From each of the four selected communities, farmers and traders will be randomly selected for the study. Systematic random sampling will be used to select households. A household list will be obtained with the assistance of community health volunteers or local authorities. Using a random start, every *nth* household will be selected. In each selected household, eligible participants, defined as women of reproductive age (with a child under five), will be invited to participate.

### Randomisation and allocation procedure

Cluster randomisation will be employed to ensure baseline comparability between the intervention and control groups for key characteristics, including age, weight, and known risk factors. The four selected farming communities will be stratified by geographic location (north and south of the district). Within each stratum, one community will be randomly allocated to the intervention group and one to the control group. Randomisation will be performed by an independent researcher using a computer-generated random number sequence. Allocation will be concealed from the field research team to reduce the risk of selection bias. Due to the nature of the intervention, blinding participants will not be feasible, as the differences between intervention and control conditions are easily observable. However, to minimise bias, the researcher responsible for delivering the intervention will be different from the researcher responsible for allocating clusters. Additionally, outcome data will be collected by individuals blinded to group assignment (44).

### Study population

The study population will be farmers, traders, women of reproductive age and children between 2 and 5 years of age in the Ho West district.

### Sample size calculation

Sample size was calculated using STATA software, accounting for a 30% (25) anticipated improvement in nutrition and reduction in waste due to the intervention. A total of 140 households (70 per group) were determined to be sufficient, assuming 80% power, a 5% significance level, and 10% non-response.

Separate sample size calculations were done for farmers and market vendors using a single proportion in STATA 14. The total sample size for these groups was set at 70, incorporating a 10% non-response rate. The estimated proportion of postharvest loss was 20%, confidence level was 0.95%, level of significance was 0.05.

### Recruitment of participants

The objective of the study will be explained to farmers, traders, and women within various households in the communities. Those who give their consent will qualify to participate in the study. The recruitment of participants will last for 1 month. To ensure adequate enrolment, the study will use community engagement through local leaders and health workers, public sensitisation via meetings and local radio, and clear communication in local languages. A multistage sampling method will ensure fair representation, while flexible scheduling and follow-up reminders will help reduce non-response and support retention.

### Inclusion and exclusion criteria

The criteria for inclusion in this study will be fruits and vegetable farmers and traders in the Ho West District.

Farmers and traders who do not deal in fruits and vegetables will be excluded, as will those who deal in fruits and vegetables outside the Ho West district. Additionally, women of reproductive age and children aged 2–5 years residing in the district will be included in the study.

**Figure 2:**
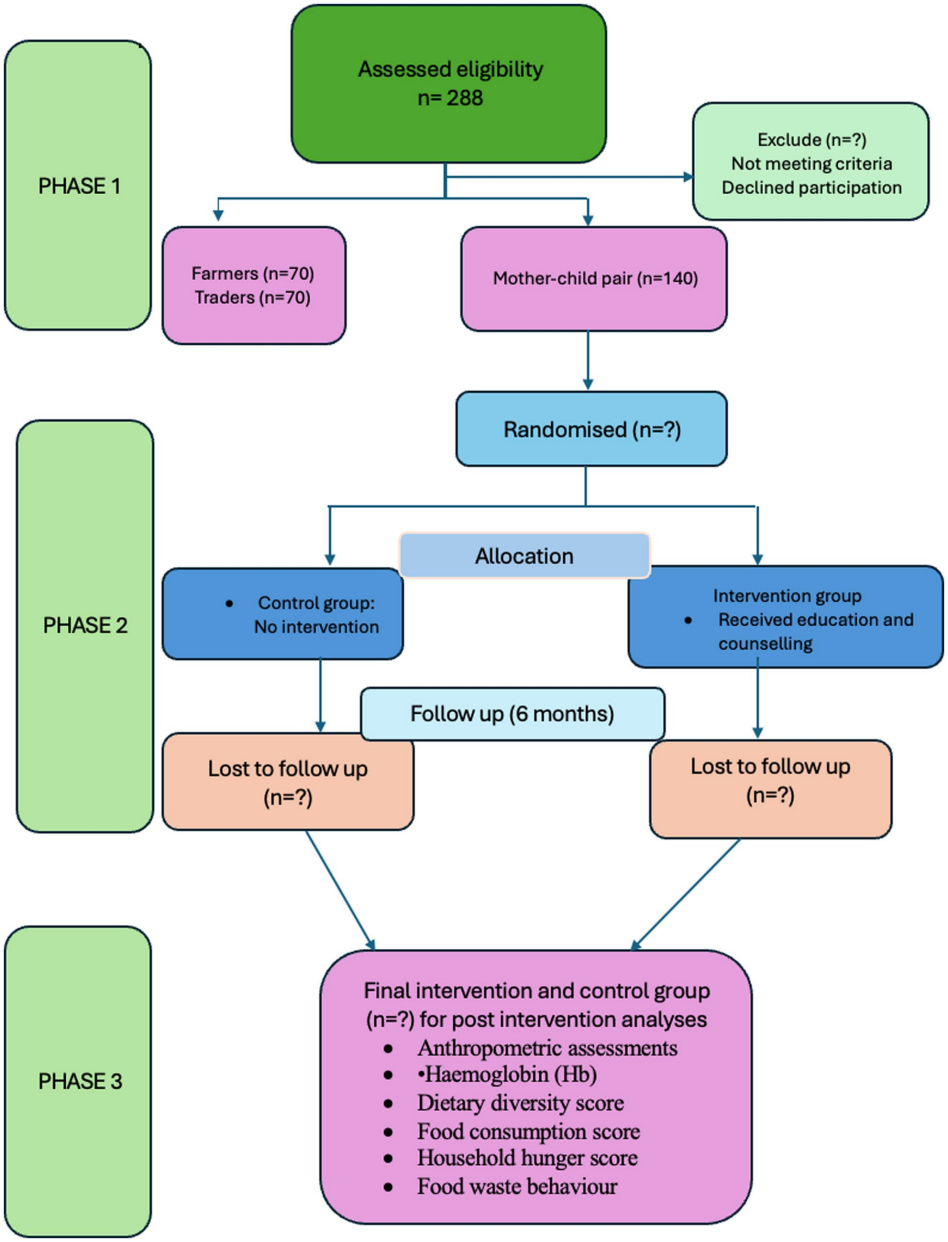
CONSORT flow chart of the study. Adapted from Moher et al. (50).

Children with congenital disorders, disabilities and diseases (Malaria, HIV, TB, cancer, severe diarrhoea, etc.) and/or requiring specialised care and hospitalisation will be excluded from the study. Children with severe malnutrition (WHZ < - −3sd or HAZ < −3sd) with complications that require inpatient care, tube-fed children, and children outside the preferred age range will also be excluded. Also, Pregnant women and women with diseases such as Tuberculosis, malaria, HIV, etc., will be excluded (Figure 2).

## Data collection

### Qualitative strand

Focus group discussions will be conducted using an interview guide developed using questions on waste and losses of fruits and vegetables at the farm, market and households, as well as factors that influence these losses. Each interview will be tape-recorded and is expected to last between 60 and 90 min.

Focus group discussions will be facilitated by a trained interviewer and assisted by a note-taker. The facilitator will lead the groups for discussions. Each focus group discussion (FGD) will last approximately 90 min. Interviews will be tape-recorded, and detailed notes will also be taken. The focus group discussion will comprise 10 farmers, traders, and women, each from the control and intervention groups.

### Quantitative strand

#### Sociodemographic characteristics

Research assistants will collect data on the socio-demographics of participants, including age, sex, level of education, occupation, income, and marital status.

#### Baseline assessment

Data will be collected using semi-structured questionnaires, a widely applied method for exploring household-level food storage and nutritional behaviours in community-based research (26, 27). The availability of fruits and vegetables, as well as their storage conditions, will be systematically observed during household visits. A structured questionnaire will be administered to gather information on sociodemographic characteristics, the quantity of fruits and vegetables purchased, consumed, stored, and the extent of food losses encountered, approaches consistent with established value-chain assessment protocols (28). Based on the quantities of reported losses, the loss estimates will be calculated using the Food Loss and Waste Value Calculator developed by Quantis as part of the WBCSD’s FReSH program (29).

## Nutritional outcomes assessment

### Anthropometric measurements

Anthropometric data—including height, weight, and Body Mass Index (BMI) for women, and z-scores for children—will be collected using standardised procedures as outlined by recent World Health Organisation guidelines (9, 42). Duplicate measurements will be recorded for each participant to ensure accuracy, and the average of the two will be used for analysis. Adult participants who can stand unassisted will be weighed barefoot and wearing minimal clothing using a calibrated Seca 876 digital scale. For infants and children unable to stand, measurements will be taken using a Seca 354 baby scale, with children either naked or in a clean, dry diaper. BMI will be computed as weight (kg) divided by height squared (m^2^) and categorised based on WHO classification thresholds. Z-scores (weight-for-height, weight-for-age, and height-for-age) will be generated using the WHO Anthro software to assess the nutritional status of children.

### Dietary diversity score

A 24-h dietary recall will be conducted to assess food intake. Participants will report all food and beverages consumed in the 24 h before the interview (41). Foods will be classified under ten categories based on FAO dietary diversity guidelines (30, 31):

1. Roots and tubers
2. Cereals and grains
3. Nuts and seeds
4. Meat, poultry, and fish
5. Eggs
6. Dairy
7. Dark leafy vegetables
8. Vitamin A-rich fruits
9. Other fruits
10. Other vegetables

Minimum dietary diversity will be defined as consumption of at least five out of ten food groups for women, and four out of seven for children, per WHO/FAO guidelines (9, 32). If a food group is consumed, a score of 1 will be given; otherwise, a score of 0.

A fruit and vegetable frequency questionnaire will also be used to assess the frequency of consumption of different fruits and vegetables. This structured questionnaire will be interviewer-administered and developed based on locally available produce. Women will report the frequency of fruit and vegetable consumption as: more than once daily, once a week but not daily, once a month but not weekly, or rarely/never. The same approach will be used for their children.

### Food consumption score

The food consumption score (FCS) will be determined by asking participants to recall all food and beverage consumption in the 7 days before the interview. Food groups will include: main staples, pulses, meat/fish, fruits, vegetables, milk, oil, and sugar. Consumption frequencies will be summed within each group and multiplied by group-specific weights. The weighted scores will be summed to derive the total FCS. FCS categories will be:

- 0–21: poor
- 21.5–35: borderline
- 35: acceptable

A score below 35 will be interpreted as indicative of household food insecurity.

### Household hunger scale

The Household Hunger Scale (45) will be used to assess food deprivation. This 3-question tool uses a 4-point Likert scale: 0 = never, 1 = rarely, 2 = sometimes, and 3 = often (more than 10 times in the past 30 days). The questions will assess:

1. Lack of food in the home due to lack of resources
2. Going to bed hungry
3. Going an entire day and night without food

### Haemoglobin testing

Capillary blood samples will be collected in the early morning (approx. 30°C, 80% humidity) using Hemocue® safety lancets. Procedures will follow WHO (33, 43) and Hemocue Hb 501 operating guidelines. Hands will be warmed and cleaned with 70% isopropyl alcohol. The third drop of blood (after discarding the first two) will be used to fill a microcuvette (~10 μL) for haemoglobin assessment using the Hemocue Hb 501 device. Samples will be taken from both women and children. Cuvettes not properly filled or with air bubbles will be discarded. Machines will be cleaned daily and validated using Hemotrol Level 1, 2, and 3 controls (Eurotrol E. V., Netherlands) (37, 38).

### Food waste behaviour

A structured questionnaire will assess food waste behaviour using adapted constructs from WRAP (34) and Stancu et al. (35). Variables will include:

- Self-reported fruit and vegetable waste
- Intention to reduce waste
- Attitudes (financial, personal, health)
- Storage and planning habits
- Household food preparation routines

Responses will be captured on a 7-point Likert scale. Mean construct scores will be used for further analysis.

### Body mass index (BMI)

The BMI-for-age z-score will be calculated using WHO Anthro Plus. BMI for women will be computed using WHO AnthroPlus by providing the weight and height in the software to provide the corresponding BMI (9).

### Theoretical framework

This study is designed as a pragmatic, theory-informed approach, based on principles from both the Health Belief Model (HBM) and Social Cognitive Theory (SCT) to influence individual and household food handling and nutrition-related behaviours. From HBM, the intervention addresses perceived susceptibility (e.g., risk of anaemia and malnutrition), perceived benefits (nutritional gains from reduced waste and improved diets), and self-efficacy (empowering women with practical storage and planning skills). From SCT, it leverages observational learning, reinforcement through group sessions, and environmental restructuring (e.g., improving storage practices) to promote sustained behaviour change. The design and messaging of the educational sessions were informed by prior empirical evidence in Sub-Saharan Africa showing that participatory and community-tailored education can effectively shift household nutrition and food waste behaviours (36).

### Intervention design and strategy

This study will implement a structured, community-based nutrition education and counselling program targeting women and children in the intervention communities of Kpedze and Amedzofe. In contrast, Abutia Kissiflui and Dededo will serve as control communities, with no intervention exposure. Such community-level strategies have been shown to improve food handling practices and reduce household-level losses in African settings (15). The aim is to enhance knowledge and practical skills on fruit and vegetable storage, reduce household-level post-harvest losses, and improve dietary practices.

The intervention will span a six-month period and will be coordinated by a trained dietitian, supported by three community health nurses. Educational meetings will occur monthly, and additional counselling will be provided through bi-weekly home visits and telephone interactions. Educational materials such as visual posters, handouts, and illustrative charts will be developed in local languages to ensure cultural relevance and ease of understanding. Group sessions will be interactive, allowing participants to ask questions and share experiences. Each session will last approximately 45 min and accommodate 5–10 women per group (Table 1).

**Table 1:** Lesson plan for nutrition education.

Month	Topic	Content	Rationale and literature support
Month 1	Importance of Fruits & Vegetables on Nutritional Status	Benefits, local examples, myths and facts	Dietary diversity and intake of micronutrient-rich foods like fruits and vegetables are critical for preventing undernutrition and anaemia in women and children (14). Awareness helps reshape food choices.
Month 2	Fruits and Vegetables Waste and Its Implications	Drivers, causes, prevention	Community-level losses reduce availability of key nutrients. Kitinoja and Kader (6) and Sheahan and Barrett (19) show that household knowledge on proper handling can reduce loss and improve food security.
Month 3–4	Fruit and Vegetable Preservation and Storage	Low-cost storage methods (e.g., clay pot coolers, FIFO), meal planning	Ambuko et al. (3) and Stathers and Mvumi (36) support the use of low-cost post-harvest technologies in rural African communities. FIFO principles help manage perishables better.
Month 5	Family Meal Planning & Use of Leftovers	Balancing meals, affordability, using local produce	FAO (1) showed that practical household-level meal planning improves intake consistency and reduces waste, especially among low-income households.
Month 6	Review	Reinforce key lessons, feedback from participants	Participatory reinforcement models improve retention and empower learners (Kumar et al., 2020). This month strengthens the learning cycle and evaluates uptake.

### Procedure for nutrition counselling session

Counselling sessions will be conducted privately, either in community meeting spaces or during home visits. Each session, lasting up to 30 min, will be delivered in a participant’s preferred language to ensure comprehension.

The key steps will include:

- Obtaining written or thumbprint consent from participants.
- Conducting a brief dietary assessment.
- Providing tailored guidance on post-harvest handling, appropriate storage methods, and reducing waste.
- Distributing a simple dietary guide/manual developed for the study.
- Following up with progress reviews every two to three weeks via calls and home visits.

## Outcomes

### Primary outcome

The primary outcome is to assess the impact of post-harvest loss of fruits and vegetables on the nutritional status of women and children.

### Secondary outcome

The secondary outcomes will be to evaluate the fruit and vegetable intake of women and children, post-harvest practices among farmers, traders, and women, food waste behaviour of women, BMI-for-age in women and children, the knowledge level of women regarding fruits and vegetables, and the impact of these on nutritional outcomes (Table 2).

**Table 2:** Table of primary and secondary outcomes.

Outcome type	Outcome	Measurement method	Assessment time points
Primary	Anaemia status in women and children	Haemoglobin testing (Hemocue 501 meter)	Baseline and post-intervention
Primary	Dietary Diversity Score (DDS)	24-h dietary recall and food group classification	Baseline and post-intervention
Secondary	Household food waste behaviour	Structured food waste behaviour questionnaire	Baseline and post-intervention
Secondary	Fruit and vegetable intake	Food Frequency Questionnaire (FFQ)	Baseline and post-intervention
Primary	Post-harvest handling knowledge/practices	Survey and Focus Group Discussion	Baseline
Primary	Anthropometric measurements (BMI, z-scores)	Height, weight using WHO protocol	Baseline and post-intervention

### Statistical analysis

Statistical package for social sciences (SPSS) (IBM Corp. Released 2022. IBM SPSS Statistics for Windows, Version 30.0. Armonk, NY: IBM Corp) will be used for the data analysis. The Kolmogorov-Smirnov and Shapiro-Wilk tests will be used to assess the normality of the quantitative data. A *p*-value less than 0.05 indicates that the data is not normally distributed, and vice versa. The characteristics of the study participants will be analysed using descriptive statistics. A chi-square test will be done to assess the relationship between categorical variables. Fisher’s exact test will be employed to determine the significance level. Parametric tests will be used to analyse normally distributed data, and non-parametric tests will be used to analyse data that is not normally distributed. Parametric tests will be presented in terms of mean and standard deviation, and non-parametric tests will be presented in terms of median (maximum-minimum). A t-test will be conducted to compare the mean differences of the outcomes of interest within and between study groups at baseline and post-intervention. Regression analysis will be conducted to predict the drivers of fruit and vegetable loss, adjusting for age and gender. Partial correlation will be used to assess the strength of association among the variables. All *p*-values will be significant at *p* < 0.05.

The primary analysis will follow the intention-to-treat (ITT) principle, including all participants as initially allocated, regardless of their adherence to the intervention protocol. This approach ensures an unbiased estimate of the intervention effect. For secondary per-protocol analyses, only participants who fully adhered to the intervention will be considered. To address missing data, particularly in outcome measures such as dietary assessments and anthropometry, multiple imputation methods will be employed under the assumption that data are missing at random. Sensitivity analyses will be conducted to assess the robustness of results. This strategy helps maintain the validity and reliability of findings despite potential data gaps.

To account for multiplicity in the analysis of primary and secondary outcomes, the study will employ a structured analytical approach. Primary and secondary outcomes will be pre-specified to avoid *post hoc* comparisons. Where outcomes are assessed at multiple time points (e.g., baseline and post-intervention), repeated-measures analysis or appropriate statistical corrections such as the Bonferroni adjustment will be applied to control the risk of Type I error. Subgroup analyses (e.g., by community or demographic characteristics) will be exploratory and clearly labelled as such to prevent overinterpretation. These steps ensure robust and reliable interpretation of findings while maintaining the statistical integrity of the study. This protocol was developed in consultation with a statistician. The statistician reviewed sample size estimations and the statistical analysis plan to ensure methodological soundness. This is reflected in the detailed statistical analysis section, which outlines methods for dealing with missing data, baseline comparability, and outcome estimation.

### Consent and participation

The trial will strictly adhere to the World Medical Association Declaration of Helsinki. The researchers will explain the purpose of the study, procedures, benefits, withdrawal options, confidentiality, and voluntary participation to participants and their guardians in a language they understand. Farmers, traders, women, and their children can participate in the study only after giving consent and assent, and their guardian must also provide consent by signing or thumbprinting the informed consent form.

### Adherence strategies and monitoring procedures

To ensure effective implementation of the intervention, this study will employ multiple strategies to improve adherence and monitor compliance. These include regular follow-up through bi-weekly phone calls and monthly home visits, simplified educational materials in local languages, non-monetary incentives such as recognition certificates, and participant feedback mechanisms to enhance engagement. Monitoring adherence will involve tracking attendance at educational sessions, conducting home visit assessments of storage practices, collecting monthly self-reports, and performing periodic dietary and anthropometric evaluations. These combined strategies aim to foster consistent participation, reinforce learning, and ensure reliable measurement of the intervention’s impact on nutritional outcomes.

### Adverse events

Potential challenges anticipated in the study include participant dropout, seasonal variations in fruit and vegetable availability, and possible contamination between intervention and control communities. The study incorporates regular monitoring, community engagement, and logistical planning to mitigate these risks. Additionally, potential discomfort during haemoglobin testing has been noted, with mitigation through trained phlebotomists and standard operating procedures.

### Ancillary and post-trial care

Given the low-risk nature of this nutrition education intervention, no formal provisions for ancillary or post-trial clinical care are planned. However, participants who experience any adverse effects related to study procedures, such as discomfort from blood sample collection, will be referred to the nearest health facility for appropriate care at no cost to the participant. Contact information for the principal investigator and healthcare personnel will be provided to all participants. While no financial compensation is offered for participation, all necessary precautions will be taken to minimise risk, and ethical obligations to ensure participant safety will be upheld throughout the study.

### Withdrawal

Participants may choose to withdraw from the research at any time without having to explain his/ her decision. The participant may choose not to answer any question they find uncomfortable or private. There will be no consequence, loss of benefit or care to any participant who chooses to withdraw from the study. However, some of the information obtained from participants without identifiers, before they decide to withdraw, may be modified or not used in final analysis reports and publications.

### Concomitant care and permitted interventions

During the trial, participants in both the intervention and control groups will be permitted to continue receiving their regular healthcare services, which are available in their communities, including antenatal care, child health services, and general outpatient care. However, any external nutrition-specific programs or food aid interventions targeting fruits and vegetables that are introduced in the study communities during the trial period will be considered prohibited interventions, as they may confound the study outcomes. Participants will be regularly monitored, and any involvement in such programs will be documented. Additionally, no therapeutic supplements, fortified foods, or nutrition-focused interventions outside the scope of the study will be allowed. This approach ensures that the effects measured are attributable solely to the study’s intervention on post-harvest loss reduction and nutrition education.

### Auditing of trial conduct

Formal external auditing of the trial is not planned due to the community-based nature and minimal risk profile of the intervention. However, internal monitoring will be conducted by the principal investigator and supervisory team to ensure compliance with the protocol, ethical standards, and data quality procedures. Periodic progress reports, including documentation of recruitment, adherence, and any adverse events, will be submitted to the ethics review board and the KNUST College of Science graduate school. While this monitoring is not independent of the investigators, it provides an appropriate oversight mechanism within the study’s operational context.

### Data monitoring

The nature of the trial may not need a data monitoring team, as the study supervisors will monitor the data collection and analysis processes. Periodic reports on the progress of the trial and adverse events will be submitted to the ethics review board by the researcher and supervisors.

### Data storage and management

All collected data will be anonymised using unique participant identification codes. Names and personally identifying information will not appear in any datasets. Data will be stored in password-protected computers with regularly updated antivirus software, accessible only to the research team. To ensure data integrity, the following quality assurance procedures such as double data entry for quantitative data using EpiData software to minimise errors, weekly consistency checks on a 10% random sample by the lead research assistant and monthly data audits by the principal investigator and a senior supervisor to verify protocol compliance and data completeness will be implemented. Focus group recordings will be transcribed, anonymised, and securely stored. Qualitative data will be stored separately from the code key. At study completion, anonymised datasets, analysis scripts, and the final protocol will be deposited in a secure institutional repository. Access will be granted upon a reasonable request and approval by the ethics board, if data protection and confidentiality guidelines are followed.

### Protocol version and date

This manuscript presents Protocol Version 1.0, finalised on June 9, 2025. Any future amendments to the protocol will be documented with a version number and date and reported to the ethics committee accordingly.

### Ethics and dissemination

The trial adheres to the ethical standards of the Declaration of Helsinki. Written informed consent will be obtained from all participants and/or their guardians before enrolment. Participation will be entirely voluntary, with no penalties for refusal or withdrawal. Ethical approval for the study was obtained from the Committee on Human Research, Publication, and Ethics (CHRPE) with reference number CHRPE/AP/044/25, and administrative clearance was secured from the Ho West District Assembly. The study is registered with the Pan African Clinical Trial Registry (PACTR202310582275172). Upon completion, anonymised datasets, statistical codes, and a full version of the protocol will be made publicly accessible through institutional repositories and shared upon reasonable request, ensuring transparency and compliance with ethical guidelines.

There are no restrictions on publication, and the research team is committed to transparent, timely, and responsible reporting in accordance with ethical guidelines and journal requirements. Authorship for any publications arising from this trial will follow the guidelines set by the chosen peer review journal. All individuals who make substantial contributions to the conception, design, data collection, analysis, interpretation, or manuscript drafting will be eligible for authorship and appropriately acknowledged. No professional medical writers will be employed for this study, and members of the research team will prepare all manuscripts to ensure transparency and intellectual ownership of the work.

### Public access to protocol, data and statistical code

The research team is committed to promoting transparency and reproducibility. Upon publication of the study findings, the full trial protocol, anonymised participant-level dataset, and statistical analysis code will be made available to the public upon reasonable request. These materials will be shared through institutional repositories or data-sharing platforms in accordance with data protection and ethical guidelines. Requests for access will be reviewed by the principal investigator and approved by the ethics committee to ensure compliance with confidentiality and responsible data use.

### Protocol amendments

Any significant modifications to the study protocol, such as changes to eligibility criteria, outcome measures, data collection procedures, or statistical analysis plans, will be formally documented and communicated to all relevant parties. These include the principal investigators, co-investigators, the ethics review committee (CHRPE), the Ho West District Assembly, and the Pan African Clinical Trial Registry. Updated versions of the protocol will include a new version number and date, and amendments will be reported in any resulting publications or trial reports in accordance with ethical and regulatory standards.

## Summary

The current study is grounded in the urgent need to address the dual challenges of post-harvest loss (PHL) and malnutrition, particularly in vulnerable populations such as women and children in rural Ghana. While PHL has been widely acknowledged for its economic challenges, its direct nutritional consequences remain inadequately explored in both scientific literature and policy discourse. This study was designed to respond to this gap by systematically examining how losses of nutrient-rich perishable foods, fruits and vegetables, along the value chain, compromise food availability, accessibility, and ultimately the nutritional status of women and children. This rationale is firmly rooted in the under-recognised link between food systems inefficiencies and public health outcomes.

The study offers three significant contributions. First, it aims to provide rigorous scientific evidence on the extent and drivers of PHL and their association with dietary diversity, food consumption scores, household hunger levels, haemoglobin concentrations, and anthropometric outcomes. Second, it introduces an integrated, community-based educational intervention aimed at improving knowledge and practices regarding post-harvest handling and consumption of fruits and vegetables. Third, the study contributes to policy development by generating context-specific, evidence-based recommendations for local and national decision-makers, agricultural stakeholders, and public health practitioners. These recommendations will support more resilient and nutrition-sensitive food systems.

The findings are expected to influence practice through the development of scalable educational models and post-harvest management strategies. On a policy level, results will provide an empirical basis for aligning agricultural and nutrition policies, especially in contexts where food loss directly undermines nutrition outcomes. The study also fills a crucial knowledge gap by demonstrating the direct link between food loss and micronutrient deficiency risks among women and children. Ultimately, this research supports the broader global agenda of sustainable food systems transformation, offering an interdisciplinary approach that integrates agriculture, nutrition, and public health for improved outcomes.

### Strengths of the study

This study offers a robust and contextually grounded investigation into post-harvest losses across the fruit and vegetable value chain in Ghana, with an emphasis on their nutritional implications. Employing a multistage sampling approach and integrating data from farmers, traders, and households will ensure representativeness and depth. The use of validated protocols for anthropometry, haemoglobin testing, and dietary assessments strengthens methodological rigour. Importantly, the study integrates context-specific dietary analysis by estimating nutrient intakes using the Ghanaian Food Composition Table, thereby ensuring cultural and dietary relevance and improving the accuracy of nutrient estimations. The inclusion of both anthropometric and biochemical assessments further strengthens the robustness and multidimensionality of the nutritional outcomes evaluated. Finally, the focus on women and children enhances the study’s public health relevance.

### Limitations of the study

This study has several limitations that should be considered. First, although the longitudinal design allows for tracking changes in fruit and vegetable waste and nutritional outcomes only at the household level over time, the follow-up period may not fully capture long-term effects. It is recommended that future studies extend the observation period to explore sustained impacts for all actors. Additionally, the assessment of fruit and vegetable waste relies on participants’ recall, which may introduce recall bias due to under- or overestimation. Finally, the study’s focus on a single district in Ghana may limit its generalizability to other regions with different agricultural practices, socioeconomic conditions, and seasonal availability of fruits and vegetables. Future research should include multiple districts to strengthen external validity and enable comparative analysis.
